# Biodegradable nano black phosphorus based SDF1-α delivery system ameliorates Erectile Dysfunction in a cavernous nerve injury rat model by recruiting endogenous stem/progenitor cells

**DOI:** 10.1186/s12951-023-02238-x

**Published:** 2023-12-18

**Authors:** Qingfeng Fu, Lujie Song, Jitao Li, Bocun Yi, Yue Huang, Zhihong Zhang, Zhongcheng Xin, Jianqiang Zhu

**Affiliations:** 1 Department of Urology, Tianjin Institute of Urology, The Second Hospital of Tianjin Medical University, Tianjin, 300211 China; 2 Department of Urology, Shanghai Jiao Tong University Affiliated Sixth People’s Hospital, Shanghai, 200233 China; 3 Department of Urology, Anqiu People’s Hospital, Weifang, Shandong 262100 China

**Keywords:** Erectile dysfunction, Endogenous stem/progenitor cells, Black phosphorus, Stromal cell-derived factor 1-α, Cavernous nerve injury.

## Abstract

Stem cell (SC) therapy has been shown high prospects in erectile dysfunction (ED) treatment. Without ethical issues and risks of immune rejection and tumorigenesis of exogenous SC therapy, endogenous stem/progenitor cells (S/PCs) have a better potential for ED management, and their homing and redistribution are controlled by SDF1-α/CXCR4 axis. Considering black phosphorus nanosheet (BPNS) has emerged as an efficient and safe drug vehicle due to its large surface area, biodegradability, and the ability to retain and slowly release its loaded drugs, BPNS is utilized to load SDF1-α, a chemokine for S/PCs, to construct the BP@SDF1-α complex to efficiently recruit stem cells (SCs) by injury-site injection and thus ameliorate ED within the bilateral cavernous nerve injury (BCNI) rat models. We find that BP@SDF1-α can efficiently recruit exogenous SCs and endogenous S/PCs to corpus cavernosum and main pelvic ganglion (MPG) by local administration. Of note, ascribing to endogenous S/PCs recruitment, it also successfully alleviates ED in BCNI rat models by enhancing the protein expression levels of α-SMA, CD31, and nNOs, and eliciting less collagen deposition in the penis after its combined injection at corpus cavernosum and MPG. Thus, this study provides a new insight into the treatment of ED with endogenous S/PCs.

**Biodegradable Nano Black Phosphorus based SDF1-α delivery system ameliorates Erectile Dysfunction in a cavernous nerve Injury Rat Model by recruiting endogenous Stem/Progenitor cells:**

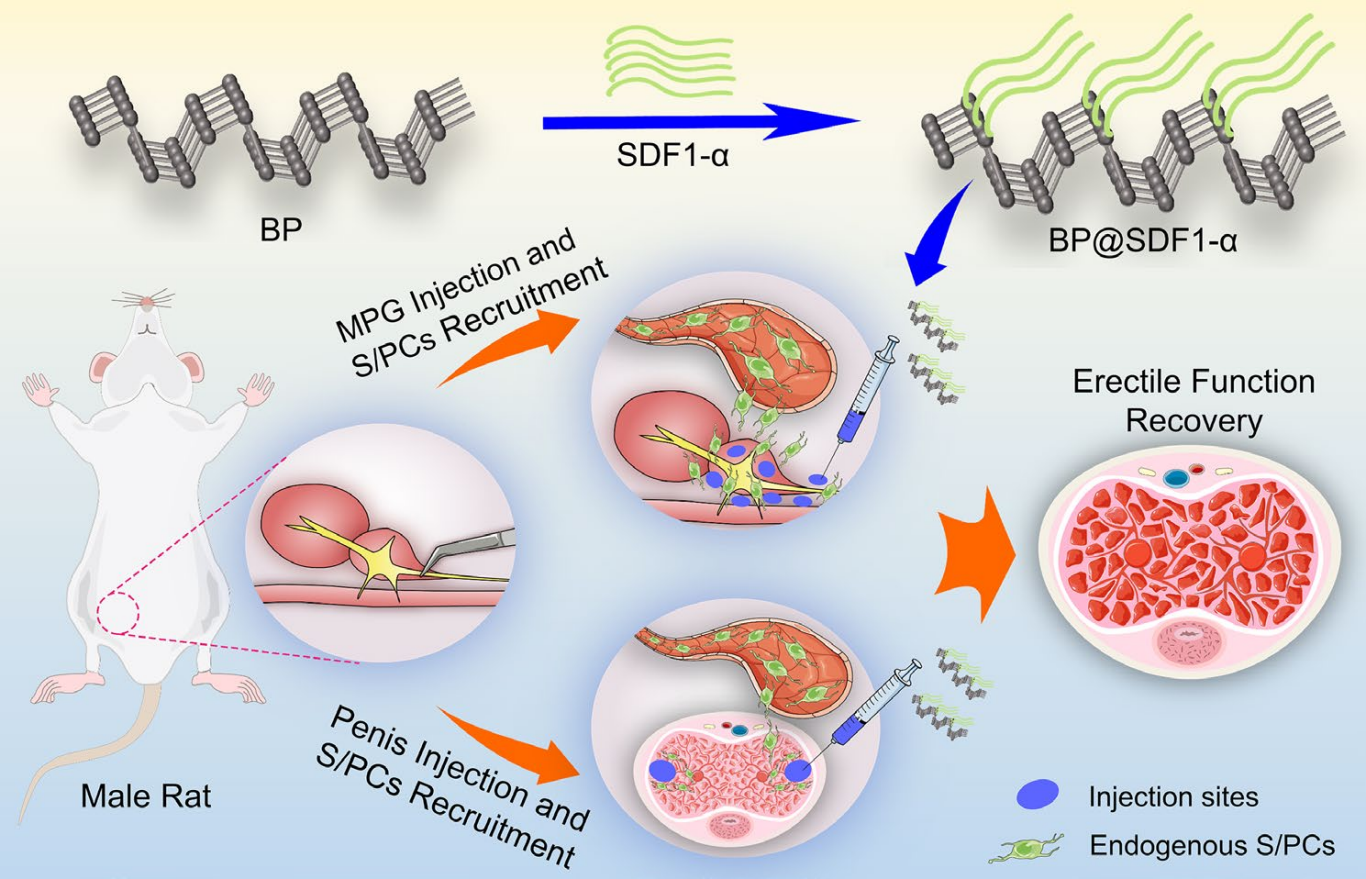

**Supplementary Information:**

The online version contains supplementary material available at 10.1186/s12951-023-02238-x.

## Introduction

ED is a major health problem affecting the life quality of more than 150 million men worldwide, and this number is predicted to reach approximately 322 million by 2025 [1, 2]. Although substantial advances have been achieved in the pathophysiological mechanisms of ED, ultimately, it seems few effective therapies for various clinical cases, especially for cavernous nerves (CNs) injury related ED [3–5]. To address this issue, various cutting-edge therapeutic strategies have been investigated and one of which is currently being extensively evaluated is SC therapy [3]. Attracted by various signals released by injured tissues, such as growth factors and cytokines, SCs can migrate to the injured tissue [6]. SCs were reported to repair injured tissues by cell migration, paracrine signaling and cytokines secretion, immunomodulation, extracellular matrix production, and differentiation and cell replacement. Their promising capacity to rescue ED was illustrated in different animal models with or without the synergy effects of cytokines or delivery platforms mainly due to paracrine signaling, cytokines secretion and differentiation of SCs [7, 8]. However, the SCs adopted in the previous studies were exogenous and their ethical and regulatory issues, insufficient sources, and potential carcinogenicity are major hindrances to their applications in clinics [9]. However, the endogenous S/PCs resident in organs and tissues possess the self-healing capability to repair damaged tissues or organs without the aforementioned issues, thus, it is an ideal SCs source to alleviate ED [9].

Numerous studies have shown that endogenous S/PCs exist in most mammalian tissues, including penile tissues [10]. However, they are scattered in penile tissues [11], which curbs their regenerative potency for damaged cavernous tissues. Therefore, it is necessary to explore an efficient endogenous S/PCs therapy for erectile function repair. In recent years, the stromal cell-derived factor 1-α/ C-X-C motif chemokine receptor 4 (SDF1-α /CXCR4) axis has attracted much attention for its ability to recruit and redistribute SCs during the repair process of damaged tissues. SDF1-α exerts its SCs recruitment function mainly through the interaction with CXCR4, a significant GTP-coupled transmembrane receptor [12]. Thus, we hypothesize that elevated local concentration and residence time of SDF1-α could efficiently recruit endogenous S/PCs to the injured site to boost the regeneration of damaged erectile function related tissues.

However, SDF1-α, as a cytokine with a small molecular weight (8–10 Kd), is easily degraded and destined to be taken away immediately by blood circulation post local injection, which limits its supposed function and application to recruit endogenous S/PCs to injured sites. Considering the unique advantages of artificial nanomaterials in drug loading and transporting, BPNS was singled out as an SDF1-α delivery platform in this study on account of its good biocompatibility, large surface area, and biodegradability in vivo [13, 14]. In this study, we constructed a BP@SDF1-α nano-drug delivery system by loading SDF1-α on the surface of BPNS and then administered it *via* corpus cavernosum and MPG local injection within BCNI rat models. By increasing local concentration and expanding the residence time of SDF1-α in corpus cavernosum, endogenous S/PCs were efficiently recruited to repair injured tissues post CNs injury, thus, improving erectile function. The results showed that BP@SDF1-α efficiently recruited endogenous S/PCs to corpus cavernosum in a time-dependent manner by local administration, and then increased the protein expression levels of α-SMA, CD31, nNOs, and reduced collagen deposition in the penis tissue. Finally, BP@SDF1-α administration improved the erectile function of BCNI rat models.

## Results

### Construction and characterization of BP@SDF1-α

**Fig. 1:**
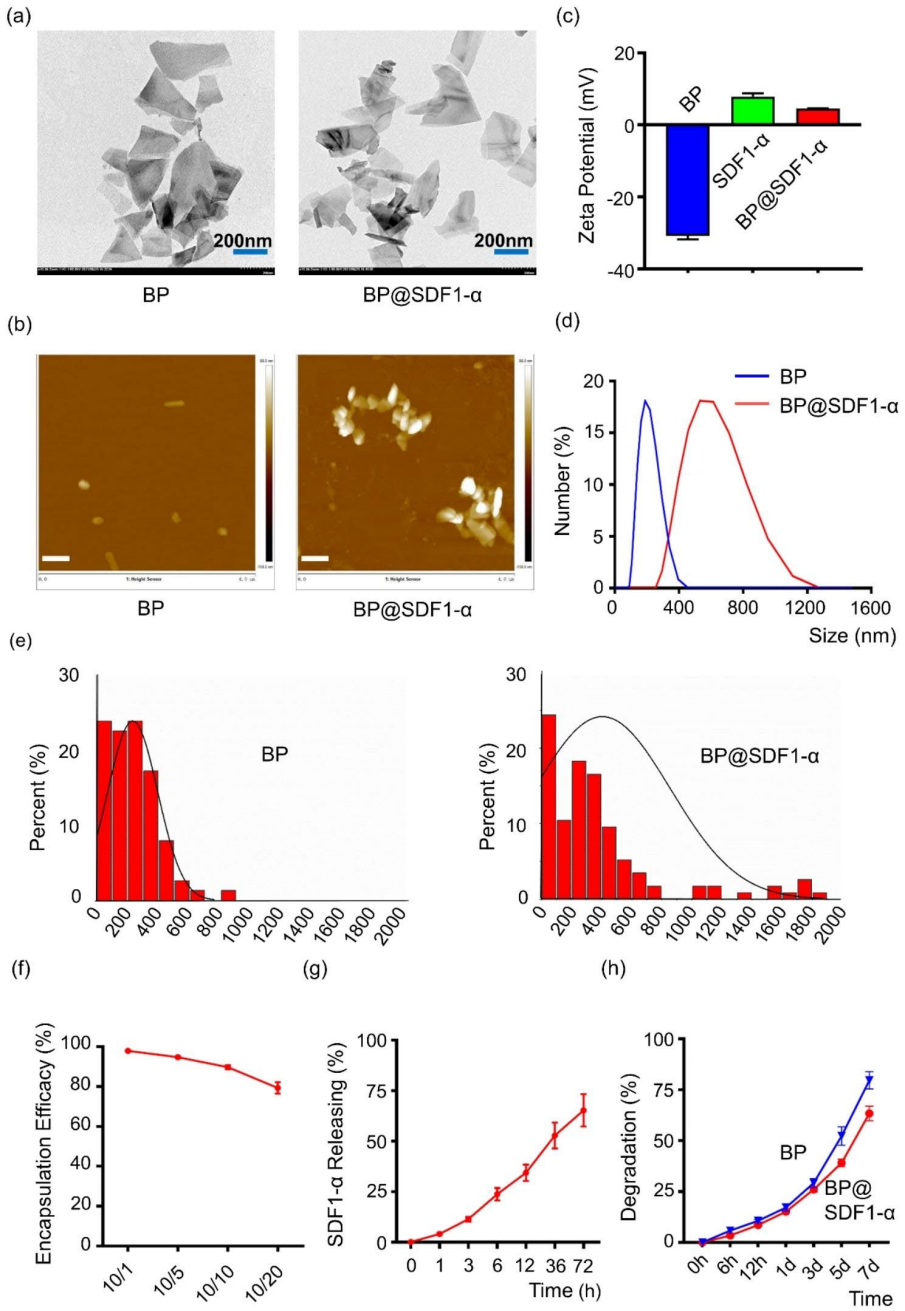
Characterization of BP. (**a**) Representative TEM images of BP and BP@SDF1-α. Scale bar, 200 nm. (**b**) Representative AFM images of BP and BP@SDF1-α. Scale bar, 500 nm. (**c**) Zeta potential of BP, SDF1-α and BP@SDF1-α in ddH_2_O. (**d**) Hydrodynamic size BP and BP@SDF1-α in ddH_2_O. (**e**) The size distribution of BP and BP@SDF1-α based on the AFM analysis by counting 100 sheets for each sample with a Gaussian fit curve (n = 100). (**f**) Encapsulation efficacy of SDF1-α at various mass ratios of BP/SDF1-α (10:1, 10:5, 10:10, and 10:20). (**g**) SDF1-α releasing curve at various time points (0, 1, 3, 6, 12, 36, and 72 h) with a mass ratio of 10:1 in ddH_2_O. (h) Degradation curve of BP and BP@SDF1-α at various time points (0 h, 6 h, 12 h, 1 d, 3 d, 5 d, 7 d) in ddH_2_O

BPNS was purchased from Shenzhen MOPHOS. Co., Ltd. (China) and similar experimental methods were processed to synthesize BP@SDF1-α [15]. Briefly, BPNS and SDF1-α were mixed and incubated at 37 °C for 30 min for the construction. BP is an ultrathin nanosheet with a lateral diameter of about 200–400 nm under transmission electron microscopy (TEM) observation and the morphology and diameter of BPNS had few changes after loading with SDF1-α (Fig. 1a). Atomic force microscopy (AFM) image showed the average lateral diameter of BPNS and BP@SDF1-α were 233.31 and 432.15 nm, respectively (Fig. 1b and e). The Zeta Potential of BPNS, SDF1-α, and BP@SDF1-α were − 30.83 mV, 7.89 mV, and 4.54 mV, respectively (Fig. 1c). After loading with SDF1-α, the hydrated particle size of BPNS increased from 198.7 to 625.8 nm (Fig. 1d). All these results indicated that SDF1-α was loaded onto the BPNS successfully. To clarify the optimal mass ratio for BP@SDF1-α construction, gradient experiments with different mass ratios (BPNS /SDF1-α) of 10:1, 10:5, 10:10, and 10:20 were conducted. BPNS showed the highest loading efficacy at the mass ratio of 10:1 and almost all the SDF1-α (around 97.87%) was loaded onto BPNS (Fig. 1f). To make the most of SDF1-α, this loading ratio was chosen to construct BP@SDF1-α for subsequent experiments. The release profile of SDF1-α from the BP@SDF1-α complex was depicted by detecting the content of SDF1-α in the supernatant (ddH_2_O). A sustained release of SDF1-α was detected and 50% SDF1-α was released from BP@SDF1-α at around 24 h (Fig. 1g). Furthermore, the stability of BPNS and BP@SDF1-α in ddH_2_O was evaluated (Fig. 1h). BP@SDF1-α showed a slower degradation process than that of BPNS which was possibly attributed to the decreased oxygen-binding area on the surface of BPNS in the BP@SDF1-α complex [16].

### Isolation and identification of adipose-derived stem cells (ADSCs)

**Fig. 2:**
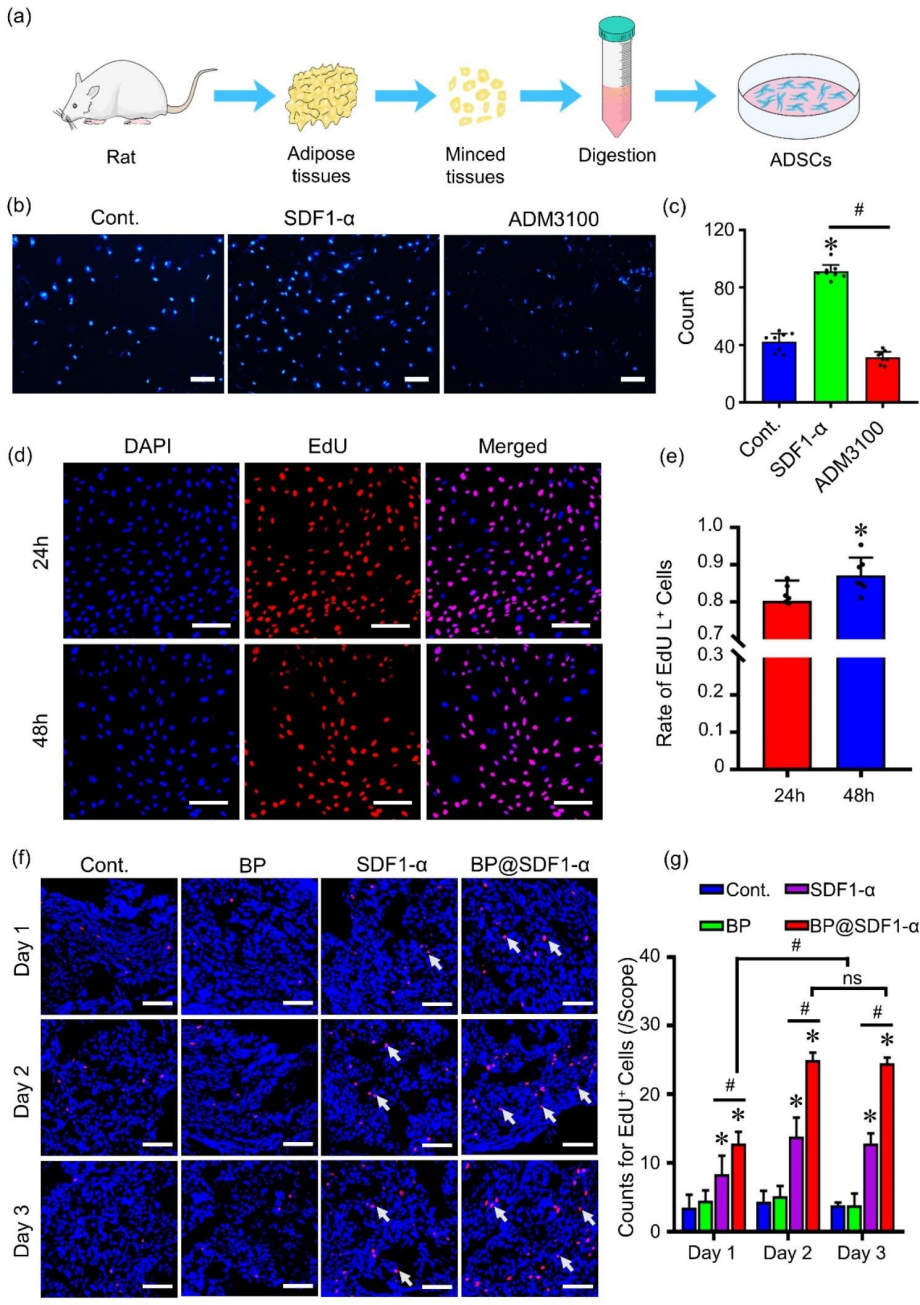
Characterization and tracking of ADSCs. (**a**) A schematic of the isolation of ADSCs. ADSCs isolated from perirenal adipose tissues of rats were cultured in vitro. (**b**) The recruitment effect of SDF1-α on ADSCs *via* SDF1-α/CXCR4 axis was tested by Transwell assay (ADM3100, a CXCR4 antagonist to block SDF1-α/CXCR4 signal transduction; nucleus in blue), and the quantified data was presented in (**c**) (n = 8, * indicated P < 0.05, compare to the Cont. group, # indicated P < 0.05). Scale bar, 100 μm. (**d**) ADSCs were labeled by incubating with EdU for various times (24 and 48 h) and the labeling efficacy of ADSCs was assessed by EdU staining (EdU positive in red, nucleus in blue), and the quantified data was presented in (**e**) (n = 7, * indicated P < 0.05). Scale bar, 200 μm. (**f**) The tracking and recruitment of ADSCs were conducted in vivo. ADSCs incubated with EdU for 48 h were harvested and injected into the penises of 8-week-old rats with various regents (PBS, BP, SDF1-α, and BP@SDF1-α, the dose of BPNS and SDF1-α were equal to BP@SDF1-α). The detection of EdU positive cells in penis tissues at different time points (1 d, 2 d, and 3 d) was visualized by laser confocal microscope (EdU positive in red, nucleus in blue), and the quantified data was presented in (**g**) (n = 6, * indicated P < 0.05, compared to the PBS group, # indicated P < 0.05, ns means no significance). Scale bar, 200 μm

Recently, mesenchymal stem cells (MSCs) have been taken as the priority in SC-based treatments in regenerative medicine, as they are less immunogenic than other types of SCs (expressing low VHL) and potentiate to escape from the immune system of the recipients [17–19]. Notably, ADSCs, a common type of MSCs, have been widely investigated because of the edge of a wide range of tissue sources and easy extraction [20]. Hence, ADSCs were selected in this study to evaluate the ability of BP@SDF1-α to recruit SCs. ADSCs were extracted from the perirenal adipose tissues of Sprague Dawley (SD) rats, and the general procedure was shown in Fig. 2a. Observed with a microscope, isolated cells presented a spindle shape and vortex arrangement at passage (P) 4 when they reached about 90% confluence.

(Additional file1: Fig. S1a). To identify the cell phenotypes, extracted cells at P4 were incubated with different cell surface markers and detected by a flow cytometer. The results indicated that these cells were strongly positive for SCs marker CD29 (97.9%), CD90 (87%), and CD44 (86.3%), while 99.23% and 98.44% of those cells were negative for endothelial marker CD31 and hematopoietic marker CD45, respectively (Additional file1: Fig. S1b), which was consistent with the markers of ADSCs in previous studies [21, 22]. As reported, ADSCs have the potential to differentiate into neurogenic, osteogenic, chondrogenic, or adipogenic lineages [23]. Similarly, the neurogenic differentiation potential of ADSCs was tested as a previous study described [24]. ADSCs presented with neuronal-like morphology after being treated with isobutylmethylxanthine (IBMX), indomethacin, and insulin for 2 days (Additional file1: Fig. S2a). Neural marker expressions of differentiated cells were examined and the results showed that these cells expressed higher levels of neuron-specific enolase (NSE), growth-associated protein 43 (GAP43), and S100 calcium-binding protein β (S100 β) than ADSCs (Additional file1: Fig. S2b), which was consistent with previous studies [25, 26]. Collectively, these results demonstrated that these extracted cells from perirenal adipose tissues were ADSCs and had the potential for neurogenic differentiation.

### BP@SDF1-α recruits ADSCs to the corpus cavernosum effectively

To confirm the SCs recruitment ability of SDF1-α, the effect of SDF1-α on ADSCs migration was tested by Transwell assay in vitro. Similar to the previous study [27], the results showed that 200 ng/mL SDF1-α significantly promoted the migration of ADSCs, which, however, was suppressed dramatically when ADM3100 (200 ng/mL, an antagonist of CXCR4) was pre-added into the Transwell system (Fig. 2b and c). The result demonstrated that SDF1-α had a good recruitment efficiency on ADSCs. To explore whether BP@SDF1-α still possesses the ability to recruit ADSCs in vivo, exogenous ADSCs were pre-labeled with 5-ethynyl-2-deoxyuridine (EdU) in vitro and visualized under a confocal microscope as a previous study reported [28]. Concretely, EdU at different concentrations was exposed to ADSCs to assess the cytotoxicity (Additional file1: Fig. S3) and its nontoxic dose of 1 µM was adopted for the detection of labeling efficiency on ADSCs at different time points *via* microscopy (Fig. 2d). ADSCs treated with EdU for 48 h had a higher labeling rate (~ 87.17%) than those with 24-hour treatment (~ 80.31%) (Fig. 2e).

Next, the BCNI rat model was constructed and 4 groups (Cont., BP, SDF1-α, and BP@SDF1-α group) of rats received intra-cavernous injection immediately with PBS, BPNS, SDF1-α, and BP@SDF1-α, respectively. Before that, ADSCs pre-treated with EdU for 48 h were harvested and injected into the corpus cavernosum of all rat models. The penises of rat models were harvested at different time points (1, 2, and 3 days) and stained with EdU detection kit. As shown in Fig. 2f and g, compared with Cont. group, the number of EdU positive cells increased to approximately 2.4-, 3.2- and 3.3-fold in the SDF1-α group and approximately 3.7-, 5.8- and 6.4-fold respectively in BP@SDF1-α group at 1, 2, and 3 days post SDF1-α and BP@SDF1-α treatment, respectively. Notably, BP@SDF1-α showed a higher efficiency in exogenous ADSCs recruitment within corpus cavernosum than SDF1-α.

### BP@SDF1-α recruits endogenous S/PCs to the corpus cavernosum and MPG effectively

**Fig. 3:**
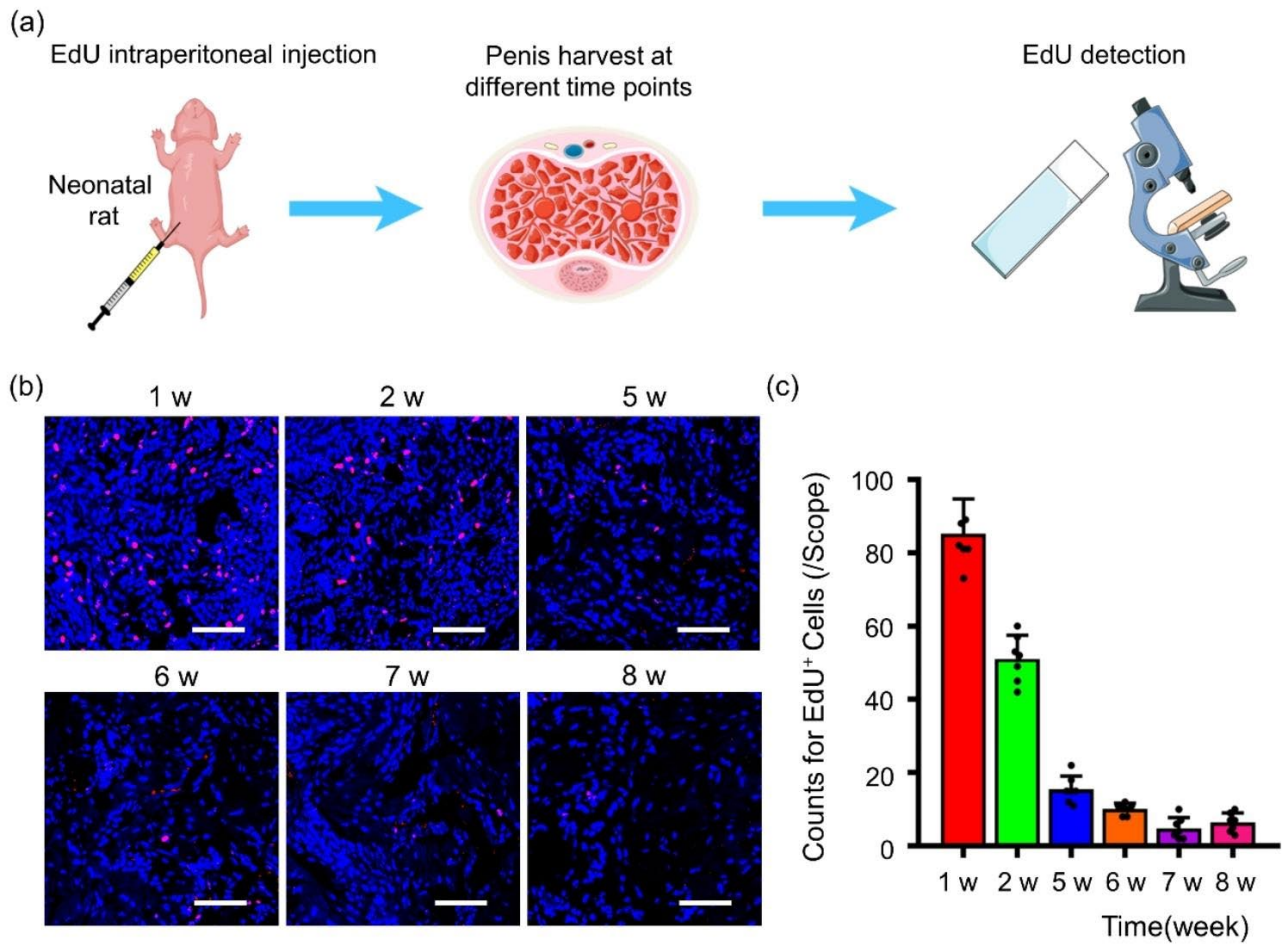
Characterization and tracking of endogenous S/PCs. (**a**) A schematic of the labeling of endogenous S/PCs. Endogenous S/PCs were labeled by intraperitoneal injection of EdU (50 mg/Kg) into new-born rats. (**b**) Endogenous S/PCs were tracked in corpus cavernosum at different time points (1, 2, 5, 6, 7, and 8 weeks post injection) by EdU staining, the EdU positive cells were visualized under laser confocal microscope (EdU positive in red, nucleus in blue), and the quantified data was presented in (**c**) (n = 6). Scale bar, 200 μm

Taking the cue that BP@SDF1-α could effectively recruit exogenous ADSCs to the corpus cavernosum, we hypothesized BP@SDF1-α also had a similar recruitment effect on endogenous S/PCs. To assess the recruitment effect of BP@SDF1-α in vivo, we utilized EdU to label and track endogenous S/PCs. EdU is a thymidine analog that is incorporated into the DNA of replicating cells to detect proliferative capacity. As we know, endogenous S/PCs exist in their niches in most mammalian tissues and remain quiescent in the G0 phase of the cell cycle under normal physiological conditions, which only divide in response to tissue damage and contribute to tissue regeneration [29]. These slow-cycling cells are capable of retaining thymidine analogs such as EdU over a long time and are also called “label-retaining cells” [29]. Based on this “label-retaining” theory, neonatal male SD rats received EdU at a dosage of 50 mg/Kg immediately after birth *via* intraperitoneal injection to label and track endogenous S/PCs as previously described (the scheme was shown in Fig. 3a) [30]. Penile tissues were harvested and processed into frozen slices at various time points (at the age of 1, 2, 5, 6, 7, and 8 weeks); EdU-labeled cells were observed *via* a confocal microscope. As shown in Fig. 3b and c, the number of EdU-labeled cells was approximately 85.3 per scope at the age of 1 week and that gradually decreased to around 6.4 per scope at the age of 8 weeks, suggesting that these quiescent cells could be identified in the corpus cavernosum up to the age of 8 weeks. Next, the frozen slices of penile tissues were co-stained with EdU and stem cell antigen-1 (Sca-1, a typical marker of SCs in rat penile tissue [25]) or CD44 (a typical marker of MSCs [22]) to further prove that EdU-labeled cells were endogenous SCs, and the results in Additional file1: Fig. S4 showed that some EdU-labeled cells were positive for Sca-1 and CD44 expression, which was consistent with a previous study [25]. Although there is a lack of a unique and strong marker for identifying endogenous S/PCs identification, our results confirm that the EdU-positive cells were endogenous S/PCs to some extent and provided strong evidence for endogenous S/PCs tracking in subsequent experiments.

**Fig. 4:**
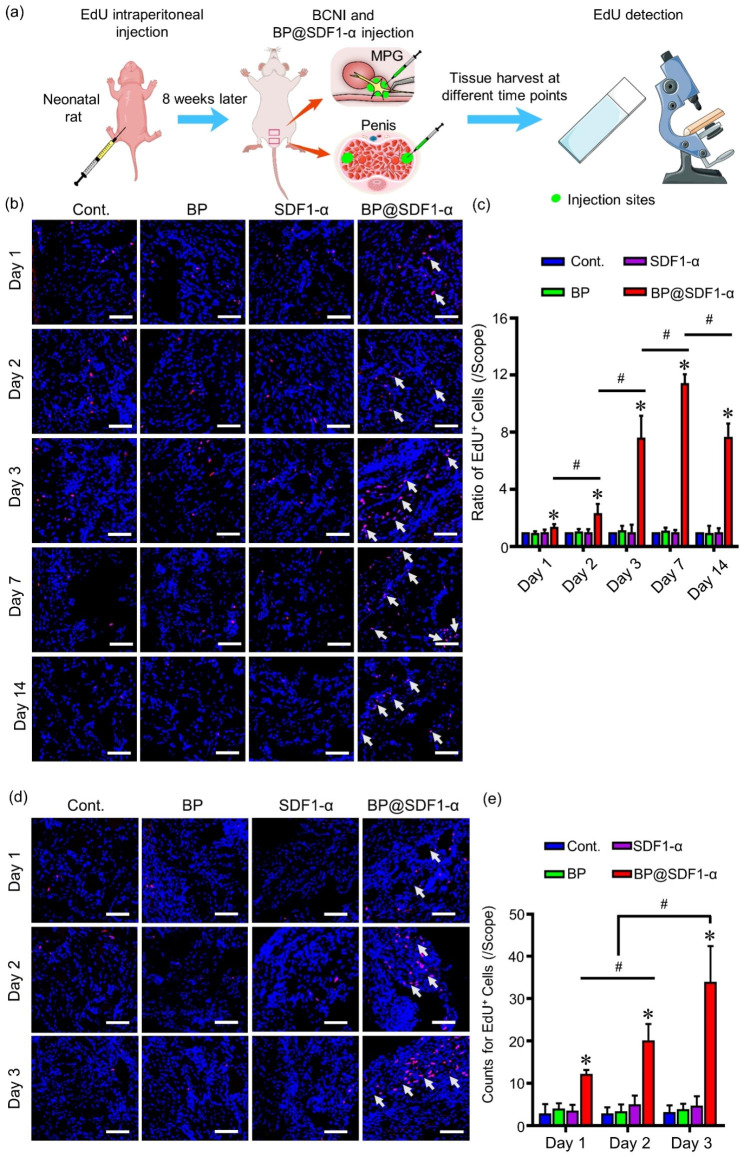
The recruitment effect of SDF1-α on endogenous S/PCs in vivo. (**a**) A schematic of detecting the recruitment of endogenous S/PCs. The recruitment effects of SDF1-α on endogenous S/PCs in corpus cavernosum and MPG were assessed. Intraperitoneal injections of EdU (50 mg/Kg) into new-born rats were conducted and various regents (PBS, BP, SDF1-α and BP@SDF1-α, the dose of BPNS and SDF1-α were equal to BP@SDF1-α) were injected into the penis and the base and periphery of MPG respectively. (**b**) The recruitment effect of SDF1-α on endogenous S/PCs in the penis was assessed. The penis tissues were harvested at different time points (1, 2, 3, 7, and 14 days post various reagents injection) and the recruitment efficacy was visualized by EdU staining of penis tissues (EdU positive in red, nucleus in blue, white arrows points to the EdU positive cells). Scale bar, 200 μm. The quantified data was presented in (**c**) (n = 6, * indicated P < 0.05, compared to the PBS group, # indicated P < 0.05). (**d**) The MPG tissues were harvested at different time points (1, 2, and 3 days post various reagents injection) and the recruitment efficacy was visualized by EdU staining of MPG tissues (EdU positive in red, nucleus in blue, white arrows points to the EdU positive cells). Scale bar, 200 μm. The quantified data was presented in (**e**) (n = 6, * indicated P < 0.05, compared to the PBS group, # indicated P < 0.05)

Similar to the ADSCs recruitment in vivo, new-born SD rats received EdU intraperitoneal injection underwent BCNI surgery at the age of 8 weeks and were randomly divided into four groups (Cont., BP, SDF1-α, and BP@SDF1-α group, 10 rats per group). The representative images of MPG and CN, CN anatomical separation and BCNI model construction were shown in Additional file1: Fig. S5a and b. The MPG and corpus cavernosum were locally injected with PBS, BPNS, SDF1-α, and BP@SDF1-α, respectively, and tissues were harvested at different time points (1, 2, 3, 7, and 14 days for corpus cavernosum and 1, 2, and 3 days post treatment for MPG) for histological examination (the scheme was shown in Fig. 4a). Compared with Cont. group, endogenous S/PCs in corpus cavernosum and MPG were recruited dramatically in the BP@SDF1-α group while there was no significant recruitment effect in the BP or SDF1-α group (Fig. 4b-e). Compared with Cont. group, the number of endogenous S/PCs in the corpus cavernosum increased to 1.4-, 2.3-, 7.6-, 11.4- and 7.8-fold at 1, 2, 3, 7, and 14 days post BP@SDF1-α treatment, respectively, and those in MPG increased to 4.3-, 7.1- and 10.7-fold at 1, 2, and 3 days post BP@SDF1-α treatment, respectively. These results illustrated our hypothesis that BP@SDF1-α local injection had a higher efficiency of endogenous S/PCs recruitment within corpus cavernosum and MPG.

### BP@SDF1-α local injection alleviates the ED in a BCNI rat model

**Fig. 5:**
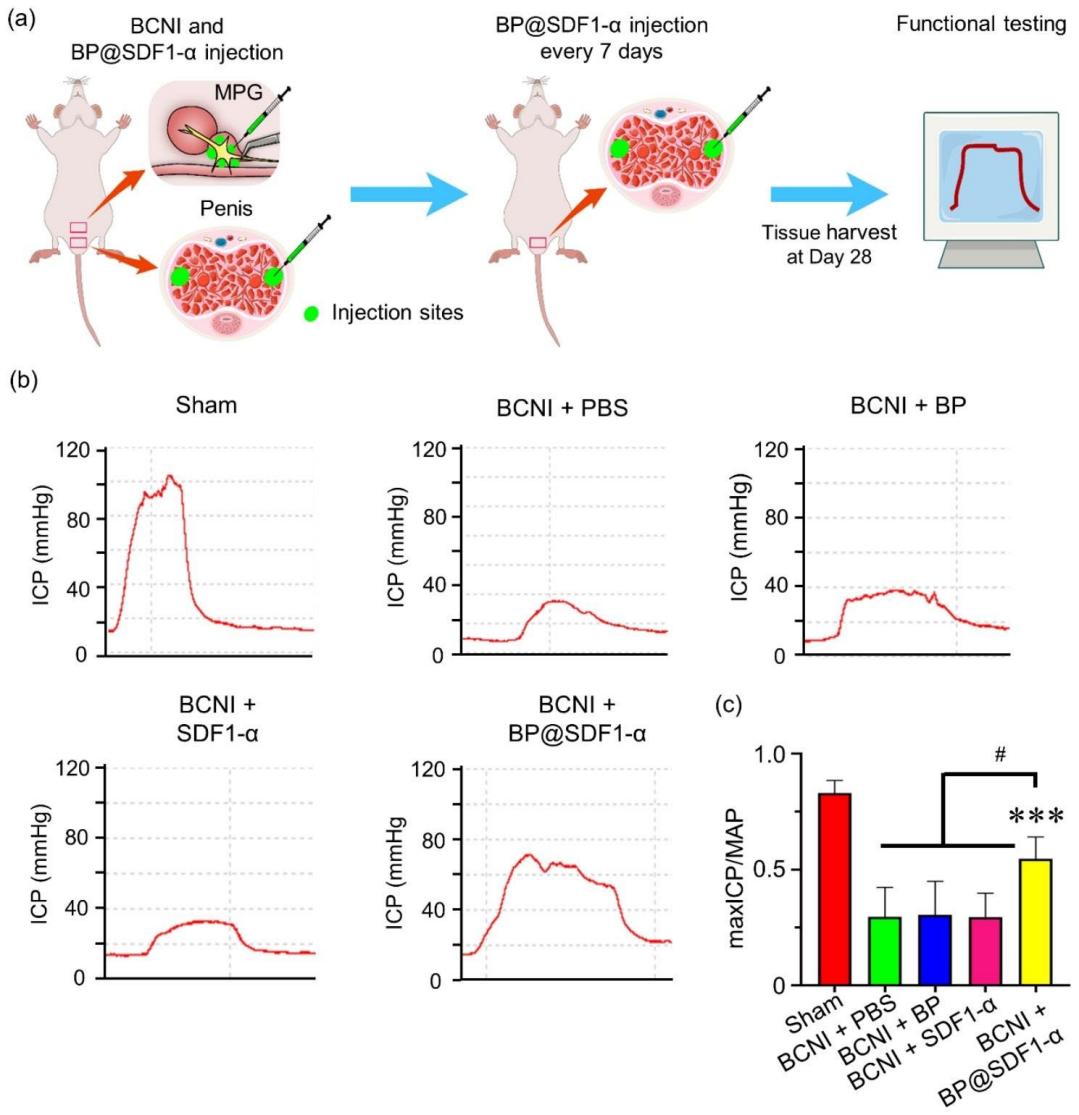
The assessment of erectile function. (**a**) A schematic of the assessment of erectile function. Eight-week-old rats with BCNI were prepared and various regents (PBS, BP, SDF1-α, and BP@SDF1-α, the dose of BPNS and SDF1-α were equal to BP@SDF1-α) were injected into the penis and the base and periphery of MPG respectively (rats in Sham group only received the same operation as BCNI rats did, without BCNI). The ICP and MAP were detected with the electric stimuli on the cavernous nerve and the penis was harvested for further tests. The ICP curves were presented in (**b**) and the quantified data of max ICP/MAP was presented in (**c**) (n = 5, *** indicated P < 0.001, compared to the PBS group, # indicated P < 0.05)

**Fig. 6:**
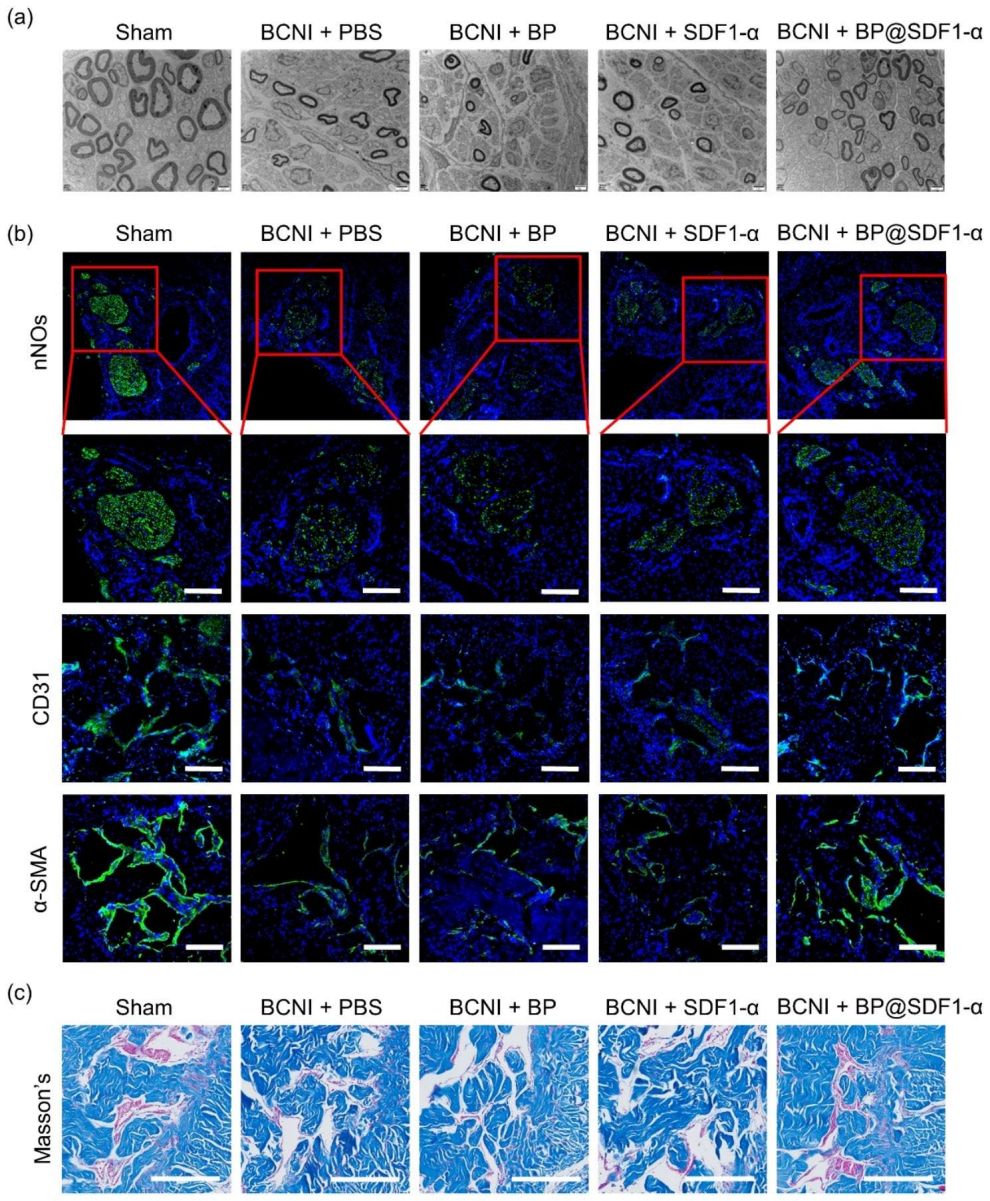
The assessment of erectile function. (**a**) The representative TEM images of cavernous nerves in different groups. Scale bars, 2 μm. (**b**) The expression of nNOs, CD31, and α-SMA in penis tissues was assessed by immunofluorescence staining and visualized under a laser confocal microscope (nNOs, CD31, and α-SMA in green, nucleus in blue), and (**c**) the representative images of Masson’s trichrome staining in different groups. Scale bar, 200 μm

Given that BP@SDF1-α could efficiently recruit endogenous S/PCs to corpus cavernosum and MPG, we reasoned that it potentiated to ameliorate ED in BCNI rats *via* endogenous S/PCs recruitment. To test the therapeutic efficacy of BP@SDF1-α on BCNI rat models, fifty 8-week-old rats were randomly divided into 5 groups (Sham, BCNI + PBS, BCNI + BP, BCNI + SDF1-α and BCNI + BP@SDF1-α group, 10 rats per group). Then, MPG and corpus cavernosum were locally injected with PBS, BPNS, SDF1-α, and BP@SDF1-α, respectively (once for MPG and every 7 days for corpus cavernosum). After the local delivery, BP@SDF1-α dramatically elevated the SDF1-α expression in penis of BCNI + BP@SDF1-α group (Additional file1: Fig. S6). Erectile function was assessed on the 28th day post-surgery in all groups (the scheme was shown in Fig. 5a). As shown in Fig. 5b and c, rats in the Sham group displayed typically normal intra-cavernous pressure (ICP) curves and the average ratio of maximum ICP to the mean arterial pressure MAP (ICP/MAP) was 0.85. BCNI surgery resulted in significantly lower ICP curves and decreased ICP/MAP ratios in BCNI + PBS group. Compared with BCNI + PBS group, rats in BCNI + BP@SDF1-α group showed significantly higher ICP curves and ICP/MAP ratio (approximately 0.52 vs. 0.30), while rats in BCNI + BP and BCNI + SDF1-α group did not display a significant difference in ICP curves and ICP/MAP ratio. The results indicated that ED was ameliorated by local injection of BP@SDF1-α. As evidenced by TEM (Fig. 6a), the number of myelin sheaths was significantly reduced after CNs injury in BCNI + PBS group. Compared with BCNI + PBS group, BCNI + BP@SDF1-α group presented a significant increase in the number of regenerated myelin, close to that in Sham group, while BCNI + BP and BCNI + SDF1-α groups showed no significant myelin regeneration. Further, immunofluorescence staining showed that the number of smooth muscle cells, endothelial cells, and dorsal penile nerves in corpus cavernosum were decreased in BCNI rats, whereas BP@SDF1-α restored their survival, upregulating the expression level of nNOs, CD31, and α-SMA in BCNI + BP@SDF1-α group (Fig. 6b). The collagen deposition in the corpus cavernosum was assessed by Masson’s trichrome staining, and the results demonstrated that the rats in BCNI + PBS group displayed much more collagen deposition than that in Sham group, while collagen deposition in BCNI + BP@SDF1-α group was significantly decreased when compared with BCNI + PBS group (Fig. 6c). Furthermore, as we know, RhoA /Rho kinase (ROCK) pathway plays a significant role in smooth muscle contraction in the corpus cavernosum and its activation is associated with ED [31]. The activation level of this pathway was detected by Western Blotting and the result showed that BP@SDF1-α downregulated the expression level of RhoA and Rock1 in rats with BCNI and that of CD31 and α-SMA were upregulated (Additional file1: Fig. S7), which was consistent with previous study [31]. Taken together, this study illustrated that BP@SDF1-α local injection could alleviate ED induced by CNs injury by efficiently recruiting endogenous S/PCs.

## Conclusion

In this study, we constructed the BP@SDF1-α delivery system to explore its ability to recruit SCs and thus ameliorate ED in a BCNI rat model. The results showed that local administration of BP@SDF1-α efficiently recruited endogenous S/PCs to corpus cavernosum and MPG in a time-dependent manner by increasing the content and prolonging the retention time of SDF1-α. Ascribing to its recruitment effect on endogenous S/PCs, BP@SDF1-α improved the erectile function of BCNI rat models by boosting the protein expression levels of α-SMA, CD31, and nNOs, and eliciting less collagen deposition in the penis after its combined injection at corpus cavernosum and MPG.

## Methods

### Preparation of BP@SDF1-α

BPNS was purchased from Shenzhen MOPHOS. Co., Ltd., China. To construct the BP@SDF1-α complex, 100 µL BPNS (100 µg/mL) aqueous solution was centrifuged and washed 3 times. Then, BPNS was suspended in 90 µL ddH_2_O, followed by the addition of 10 µL SDF1-α (100 µg in 1mL ddH_2_O, PeroTech, USA, 400-32 A). The mixture of BPNS and SDF1-α was incubated for 30 min at 37 °C after a gentle vortex. Finally, the mixture was centrifuged and dispersed in 100 µL ddH_2_O or PBS for further study.

### Characterization of BP@SDF1-α

TEM analyses of BPNS and BP@SDF1-α were performed with an H-7500 transmission electron microscope (Hitachi Scientific Instruments, Japan). AFM images were taken *via* an AFM 5500 instrument in the contact mode (Agilent Technologies, Inc., USA) at the concentration of 50 µg/mL in ddH_2_O. Raman spectra were recorded by using an InVia Raman microscope (Renishaw, UK). Zeta potential and hydrodynamic diameter measurements of BPNS materials in ddH_2_O at the concentration of 20 µg/mL were assayed with a Zeta-sizer (Malvern Nano series, Malvern, U.K.).

### Loading and releasing capacity of BP@SDF1-α

The loading and releasing capacity of BP@SDF1-α was determined by the Rat SDF1-α ELISA Kit (MEIMIAN, China, MM- 20919R1). Briefly, the mixture of BPNS and SDF1-α was incubated in ddH_2_O for 30 min at 37 °C with various mass ratios (10:1, 10:5,10:10, and 10:20). Then, the supernatant was collected after centrifugation. Finally, the concentration of free SDF1-α in the supernatant was determined. BP@SDF1-α with a mass ratio of 10:1 was constructed and resuspended in ddH_2_O. The supernatant of BP@SDF1-α complexes was collected at different time points (0, 1, 3, 6, 12, 36, and 72 h after resuspension) and the content of SDF1-α was detected by the Rat SDF1-α ELISA Kit.

### The degradation rate of BP@SDF1-α

BP@SDF1-α was constructed. Then the supernatant of BPNS and BP@SDF1-α were collected individually at different time points (0 h, 6 h, 12 h, 1 d, 3 d, 5 d, and 7 d after resuspension) and the phosphate in the supernatant was detected by Phosphate Sensor Assay Kit (Beyotime Biotechnology, China, S0192S).

### Isolation of ADSCs

All animal experiments in this study were conducted with the approval of the Institutional Animal Care and Use Committee of Yi Shengyuan Gene Technology (Tianjin) Co., Ltd. All animals were housed in an environmentally (temperature and humidity) controlled room with a 12-h light or dark cycle and free access to laboratory chow and water. Perirenal adipose tissues obtained from eight-week-old male SD rats were washed 3 times in Dulbecco’s Modified Eagle Medium: Nutrient Mixture F-12 (DMEM/F12) medium (Gibco BRL Life Technologies Inc., USA) with 1% penicillin/streptomycin (Hyclone, CA, USA) and then transferred to a petri dish. After the removal of blood vessels and fascial tissues, adipose tissues were cut into pieces (1–2 mm^3^) and digested at 37 °C in DMEM/F12 medium containing 0.1% collagenase I (Solarbio Life Science, China) for 1 h. The tissue precipitation was collected and resuspended in erythrocyte lysate (Solarbio Life Science, China) for 5 min at room temperature. After filtration through 100 μm nylon filter mesh (BD Falcon) and centrifugation, 1 × 10^6^ cells were plated on a 10-cm dish with complete DMEM/F12 medium (supplemented with 10% fetal bovine serum (FBS) (Gibco BRL Life Technologies Inc., USA) and 1% penicillin/streptomycin).

### Identification and differentiation of ADSCs

The identification of ADSCs was tested by flow cytometry analysis. Briefly, three passaged ADSCs were collected and incubated with fluorescein isothiocyanate (FITC)-conjugated anti-CD44, anti-CD90, phycoerythrin (PE)-conjugated anti-CD29, Alexa Fluor 647-conjugated anti-CD45, and Cyanine 7- conjugated anti-CD31 and their isotypic controls (all these antibodies were purchased from BioLegend, lnc.) respectively at room temperature for 30 min in dark. These cells were washed 3 times with PBS and tested by flow cytometry (Cytomics FC500 MPL, USA) and the results were analyzed with the FlowJo software. For neural differentiation, 70–80% confluent ADSCs were cultured in 10% FBS-containing DMEM/F12 supplemented with 5 µg/mL insulin, 200 µM indomethacin (INDO), and 500 µM IBMX for 48 h. The morphology changes were observed under an optical microscope and the expression of neural markers (NSE, GAP43, and S100β) was tested by Western Blotting.

### Construction of BCNI model

The procedure of BCNI mainly referred to the previous studies [32, 33], Eight-week-old rats were anesthetized with sodium pentobarbital (60 mg/Kg) and placed in the supine position on an experiment table with the lower half of the abdomen shaved. A 5 cm incision at the lower, midline abdomen was made to fully expose the prostate, MPG, and CNs. The bilateral CNs crush was performed at around 5 mm distal from the ganglion for 2 min *via* the “hemostat tip-syringe needle-nerve-hemostat tip” sandwich structure [32]. Finally, the abdomen was closed and anti-inflammatory drugs were administrated.

### Labeling and tracking of SCs

ADSCs were cultured in glass bottom cell culture dishes with a complete DMEM/F12 medium. After reaching 50% confluence, cells were incubated with EdU (Invitrogen) for 24 and 48 h respectively. Then cells were processed and stained under the instructions of Click-iT™ EdU Cell Proliferation Kit for Imaging (Invitrogen) and EdU-labeled ADCSs were visualized with a confocal microscope (Olympus GmbH, Hamburg, Germany). Eight-week-old SD rats were purchased for ADSCs tracking in vivo. All rats were randomly divided into four groups (Cont., BP, SDF1-α, and BP@SDF1-α group) and underwent BCNI surgery. Each rat was injected with EdU-labeled ADSCs (incubated with EdU for 48 h) and then MPG and corpus cavernosum were locally injected with PBS, BPNS, SDF1-α, and BP@SDF1-α, respectively. Briefly, during the BCNI procedure, an extended skin incision was made to expose the penis. The needle was inserted near the distal tip of one side of the penis. After inserting the needle at a certain distance approximately to the proximal tip, 100 µL of PBS suspended with 0.5 × 10^6^ ADSCs was slowly injected into the cavernous sinus as the needle was withdrawn. The injection site was then held under pressure for 3 min after the completion of the injection and the opposite side received the same procedure. Forty minutes later, an elastic band was placed at the base of the penis to block blood flow, similar injection method was applied to conduct bilateral intra-cavernosum injections at the sub-albuginea in different groups (injected with 100 µL of PBS, BPNS, SDF1-α, and BP@SDF1-α, respectively, 50 µL per side, 1.2 µg SDF1-α per side), and the rubber band was removed 6 minutes after the injection was completed. As for MPG local injection, 30 µL PBS, BPNS, SDF1-α or BP@SDF1-α was injected into the base and periphery of bilateral MPG in different groups (15 µL per side, 0.5 µg SDF1-α per side). After injection, a small visible bulge appeared at the site of the MPG. A small drop of tissue adhesive was then added to the injection site to prevent drug leakage when the needle was removed. All rats were kept after the surgery. The penile tissues were finally harvested at different time points (1, 2, and 3 days post treatment) for fluorescent staining.

For endogenous S/PCs, neonatal male SD rats received intraperitoneal injections of EdU at a dosage of 50 mg/Kg immediately after birth. A group of rats was sacrificed at the age of 1, 2, 5, 6, 7, and 8 weeks, the penile tissues were collected respectively and processed for EdU detection by confocal microscope. Another group of rats underwent BCNI at the age of 8 weeks and were randomly divided into four groups (Cont., BP, SDF1-α, and BP@SDF1-α group). MPG and corpus cavernosum were locally injected with PBS, BPNS, SDF1-α, and BP@SDF1-α, respectively (details were shown above). The tissues were harvested at different time points (1, 3, 7, and 14 days post treatment) for fluorescent staining.

### ICP and MAP measurement

Fifty 8-week-old rats were randomly divided into 5 groups (Sham, BCNI + PBS, BCNI + BP, BCNI + SDF1-α and BCNI + BP@SDF1-α group, 10 rats per group). Rats in Sham group were received the process of BCNI surgery without bilateral CNs crush, and rats in other groups underwent complete BCNI surgery. Subsequently, MPG and corpus cavernosum were locally injected with PBS, BPNS, SDF1-α, and BP@SDF1-α, respectively (once for MPG and every 7 days for corpus cavernosum). Twenty-eight days after BCNI, ICP and MAP were measured to evaluate erectile function as in previous studies [32, 33]. Briefly, rats were anesthetized and received laparotomy at the lower, midline abdomen to expose MPG and CNs for ICP detection and the left carotid artery was exposed for recording MAP. For ICP detection, a 24-gauge needle filled with 150 U/mL heparin solution was inserted into the left corpus cavernosum to conduct signal through a pressure transducer, while a bipolar electrode was used for stimulating the CNs with parameters of 5 mV, 20 Hz, 0.2 ms pulse width, and 60 s duration. MAP was recorded through a pressure transducer and a polyethylene-50 tube inserted into the left carotid artery.

### Transmission electron microscopy (TEM)

Approximately 2-mm nerve segments distal to the injured site of CNs in rats were harvested and quickly placed in pre-cooled 2.5% glutaraldehyde for 4 h. After the post-fixation in 1% OsO4 for 3 h, the nerve segments were embedded and cut into super-thin slices, and stained with 3% acetic acid uranium-nitrate lead. The morphology of CNs was observed under an electron microscope (H-7500, Hitachi Corp., Tokyo, Japan).

### Masson Trichrome Staining

Penile tissues were harvested from SD rats and fixed in 4% paraformaldehyde. The tissues were then dehydrated, embedded and processed into slices. These slices were stained under the instruction of a Masson Trichrome staining kit (Solarbio Life Science, China).

### Immunofluorescence staining

Slices of penile and MPG tissues were processed for immunofluorescence staining. Briefly, after the fixation with 2% formaldehyde and permeabilization with 0.5% TritonX-100, slices were incubated with primary antibodies (anti-α-SMA, anti-nNOs, anti-CD31, anti-Sca-1, and anti-CD44, ProteinTech Group, Inc) at 4 °C overnight and then incubated with CoraLite 488/594-conjugated Goat Anti-Rabbit IgG (ProteinTech Group, Inc) respectively at room temperature for 2 h in dark. Tissues were visualized under a confocal microscope (Olympus GmbH, Hamburg, Germany).

### Western blotting

Cells or tissues were harvested and lysed by RIPA lysis buffer (Beyotime Biotechnology, China) supplemented with a protease inhibitor cocktail (Solarbio Life Science, China). The concentrations of total protein were detected *via* a BCA Protein Assay Kit (Solarbio Life Science, China) and equal amounts of protein were loaded into sodium dodecyl sulfate-polyacrylamide gel electrophoresis (SDS-PAGE) and then proteins were transferred onto Nitrocellulose (NC) membrane (Pall Corporation, USA). Later, NC membranes were incubated with different primary antibodies (anti-α-SMA, anti-CD31, anti-NSE, anti-GAP43, anti-S100β, anti-RhoA and anti-Rock1, ProteinTech Group, Inc) at 4 °C overnight and then incubated with HRP-conjugated secondary antibody for 1 h at room temperature. The signals were detected with the BIO-RAD ChemiDoc XRS chemiluminescence system (Bio-Rad Inc., CA, USA).

### Statistical analysis

All data were analyzed by GraphPad Prism 8 software and presented as mean ± standard deviation. was used for the statistical analysis of data. An independent T-test or one-way ANOVA test was performed to determine differences between or among groups.

### Electronic supplementary material

Below is the link to the electronic supplementary material.

**Additional file 1**: **Fig. S1**. The identification of ADSCs. (a) Representative image of spindle-shaped ADSC in passage 4. Scale bar, 200 μm. (b) The surface antigens expression of ADSCs (CD31, CD45, CD29, CD90 and CD44). **Fig. S2** The neural differentiation potential of ADSCs. (a) Representative image of induced ADSCs cultured in neural differentiation induction medium for 24 h. Scale bar, 200 μm. (b) The induced ADSCs were harvested and the expression of neural related markers (NSE, GAP43 and S100β) was detected by Western Blotting. **Fig. S3** The cell viability of ADSCs after 48-hour exposure to EdU at various doses, * indicated P < 0.05. **Fig. S4** The identification of endogenous S/PCs. Endogenous S/PCs were characterized in penis tissues at the age of 8 weeks by immunofluorescence staining of Sca-1, CD44 (stem cell markers) and EdU staining (Sca-1 and CD44 in green, EdU positive in red, nucleus in blue). Scale bar, 200 μm. **Fig. S5** (a) Representative image of MPG and CN. (b) Representative image of CN anatomical separation (over the black suture) and the “hemostat tip-syringe needle-nerve-hemostat tip” sandwich structure for BCNI model construction (BL, bladder; PE, penis; MPG, main pelvic ganglion; CN, cavernous nerve; PN, pelvic nerve). **Fig. S6** The expression of SDF1-α in penis tissues was assessed by immunofluorescence staining and visualized under a laser confocal microscope (SDF1-α in green, nucleus in blue). Scale bar, 200 μm. **Fig. S7** The protein expression level of Rock1, RhoA, and CD31 in penis tissues was detected by Western Blotting

## Data Availability

The data that support the findings of this study are available from the corresponding author upon reasonable request.

## Abbreviations

SC: Stem cell
ED: Erectile dysfunction
S/PCs: Stem/progenitor cells
SDF1-α: Stromal cell-derived factor 1-α
CXCR4: C-X-C motif chemokine receptor 4
BPNS: Black phosphorus nanosheet
SCs: Stem cells
BCNI: Bilateral cavernous nerve injury
MPG: Main pelvic ganglion
CNs: Cavernous nerves
TEM: Transmission electron microscopy
AFM: Atomic force microscopy
ADSCs: Adipose-derived stem cells
MSCs: Mesenchymal stem cells
EdU: 5-ethynyl-2-deoxyuridine
Sca-1: Stem cell antigen-1
ICP: Intra-cavernous pressure
MAP: Mean arterial pressure
