# Supplementary material for: Biodegradable nano black phosphorus based SDF1-α delivery system ameliorates Erectile Dysfunction in a cavernous nerve injury rat model by recruiting endogenous stem/progenitor cells
Source: J Nanobiotechnology. 2023 Dec 18;21:487. doi: 10.1186/s12951-023-02238-x (PMC10726595; doi:10.1186/s12951-023-02238-x)
Supplement: Supplementary file 1 — Additional file 1: Fig. S1. The identification of ADSCs. (a) Representative image of spindle-shaped ADSC in passage 4. Scale bar, 200 μm. (b) The surface antigens expression of ADSCs (CD31, CD45, CD29, CD90 and CD44). Fig. S2 The neural differentiation potential of ADSCs. (a) Representative image of induced ADSCs cultured in neural differentiation induction medium for 24 h. Scale bar, 200 μm. (b) The induced ADSCs were harvested and the expression of neural related markers (NSE, GAP43 and S100β) was detected by Western Blotting. Fig. S3 The cell viability of ADSCs after 48-hour exposure to EdU at various doses, * indicated P < 0.05. Fig. S4 The identification of endogenous S/PCs. Endogenous S/PCs were characterized in penis tissues at the age of 8 weeks by immunofluorescence staining of Sca-1, CD44 (stem cell markers) and EdU staining (Sca-1 and CD44 in green, EdU positive in red, nucleus in blue). Scale bar, 200 μm. Fig. S5 (a) Representative image of MPG and CN. (b) Representative image of CN anatomical separation (over the black suture) and the “hemostat tip-syringe needle-nerve-hemostat tip” sandwich structure for BCNI model construction (BL, bladder; PE, penis; MPG, main pelvic ganglion; CN, cavernous nerve; PN, pelvic nerve). Fig. S6 The expression of SDF1-α in penis tissues was assessed by immunofluorescence staining and visualized under a laser confocal microscope (SDF1-α in green, nucleus in blue). Scale bar, 200 μm. Fig. S7 The protein expression level of Rock1, RhoA, and CD31 in penis tissues was detected by Western Blotting [file 12951_2023_2238_MOESM1_ESM.docx]

Supplementary Information

Biodegradable Nano Black Phosphorus based SDF1-α Delivery System Ameliorates Erectile Dysfunction in a Cavernous Nerve Injury Rat Model by Recruiting Endogenous Stem/Progenitor Cells

Qingfeng Fu^1^‡, Lujie Song^2^‡, Jitao Li^3^, Bocun Yi^1^, Yue Huang^1^, Zhihong Zhang^1^, Zhongcheng Xin^1^, Jianqiang Zhu^1^*


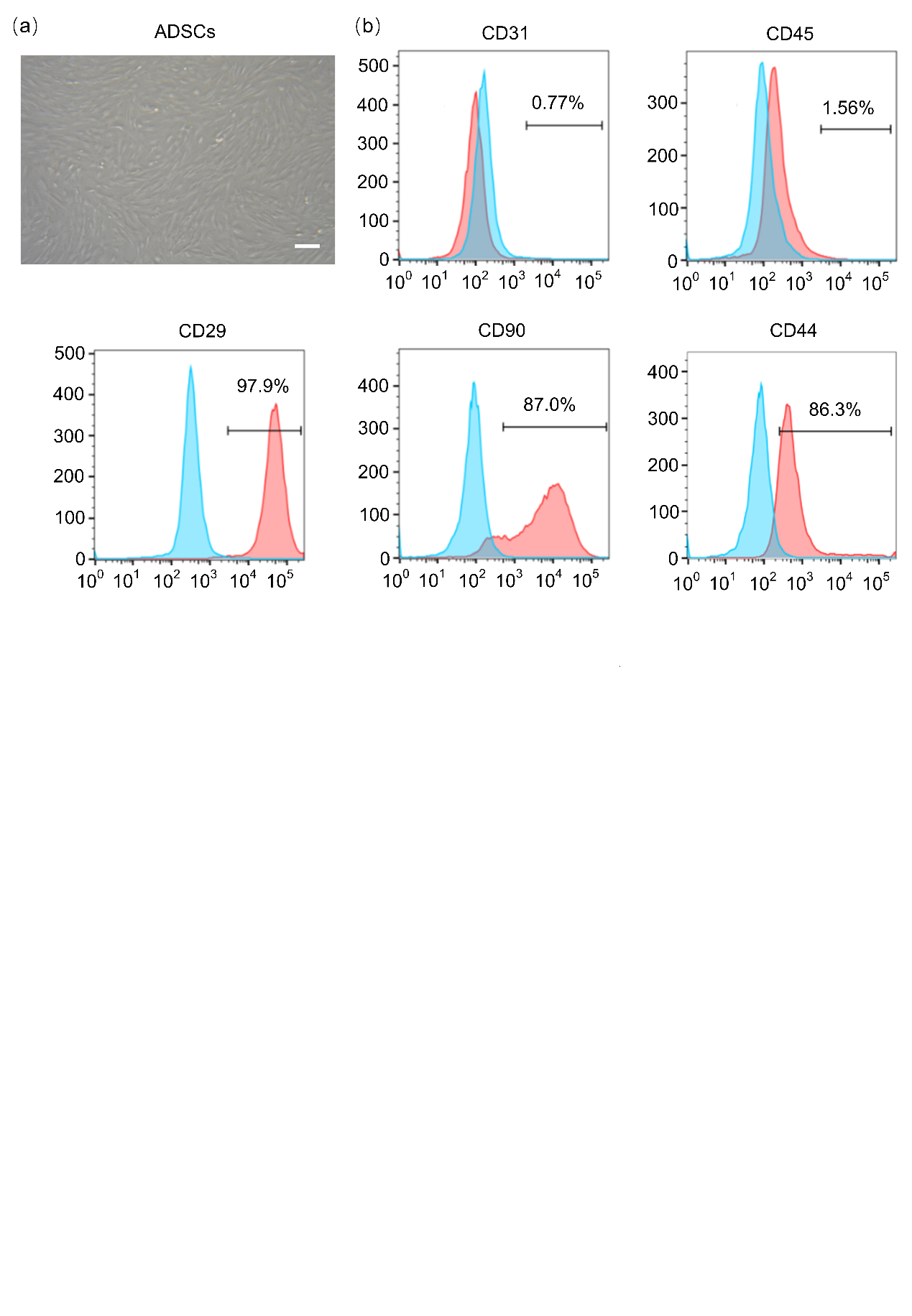


**Fig. S1** The identification of ADSCs. (a) Representative image of spindle-shaped ADSC in passage 4. Scale bar, 200 μm. (b) The surface antigens expression of ADSCs (CD31, CD45, CD29, CD90 and CD44).


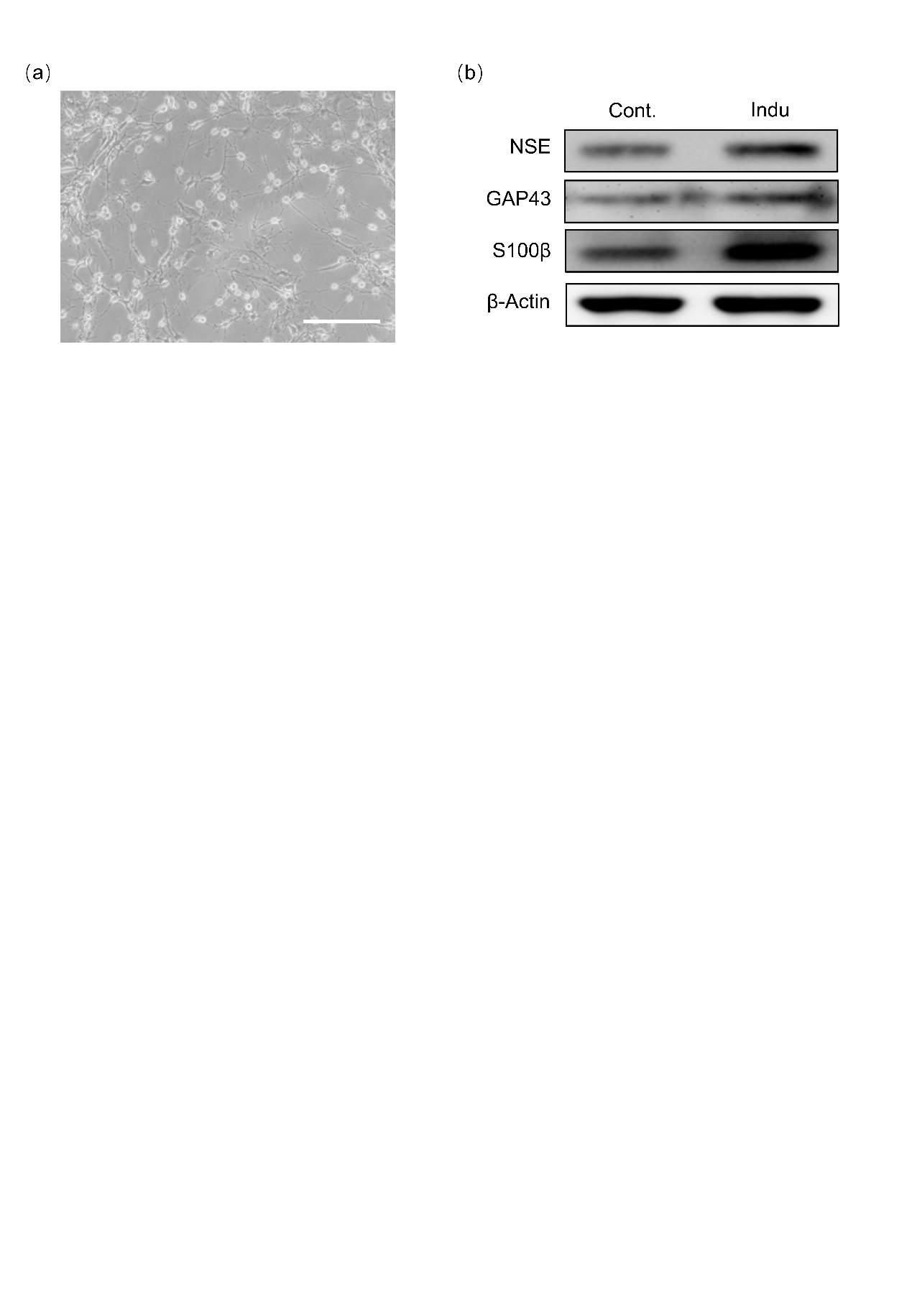


**Fig. S2** The neural differentiation potential of ADSCs. (a) Representative image of induced ADSCs cultured in neural differentiation induction medium for 24 h. Scale bar, 200 μm. (b) The induced ADSCs were harvested and the expression of neural related markers (NSE, GAP43 and S100β) was detected by Western Blotting.


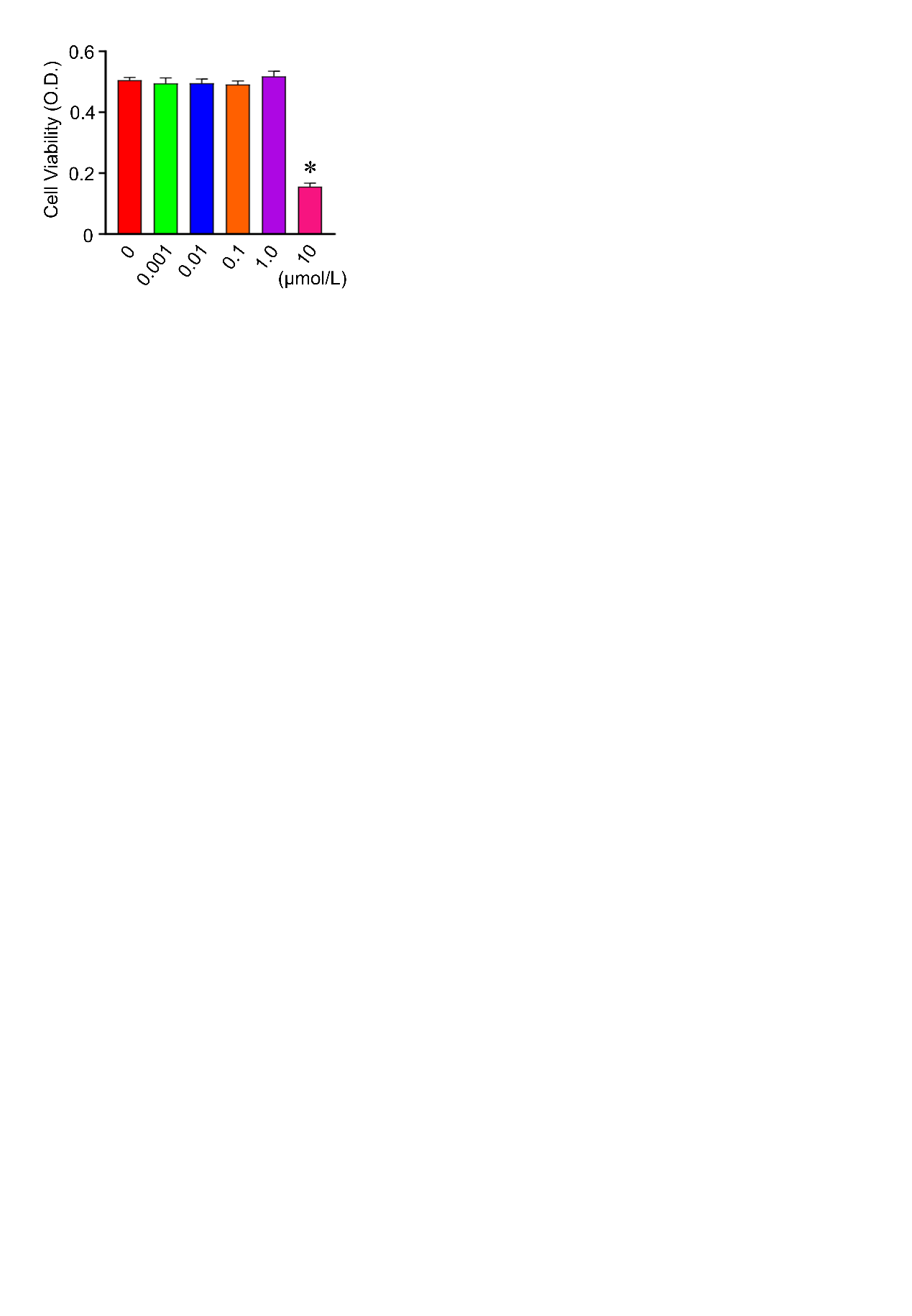


**Fig. S3** The cell viability of ADSCs after 48-hour exposure to EdU at various doses, * indicated P < 0.05.


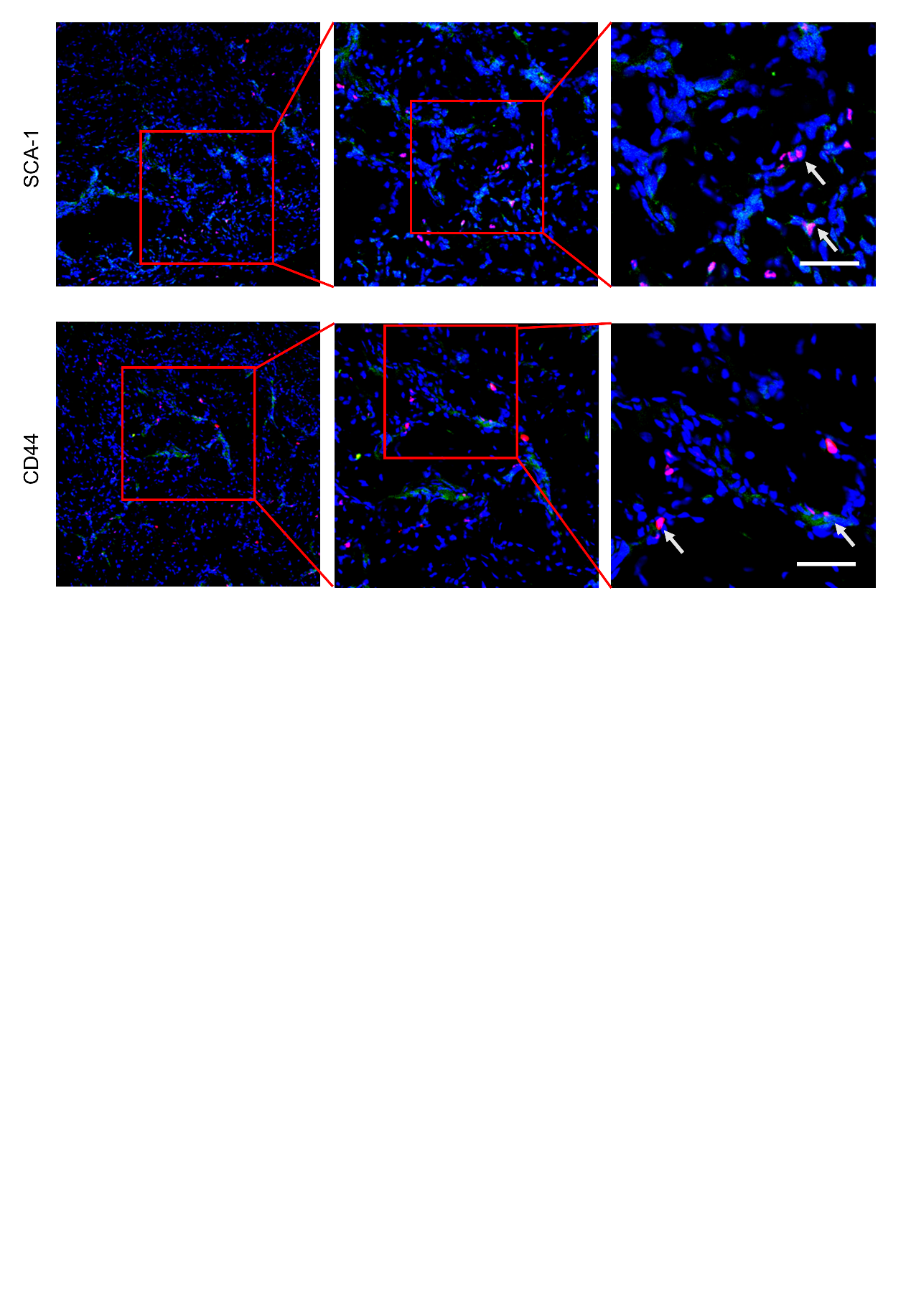


**Fig. S4** The identification of endogenous S/PCs. Endogenous S/PCs were characterized in penis tissues at the age of 8 weeks by immunofluorescence staining of Sca-1, CD44 (stem cell markers) and EdU staining (Sca-1 and CD44 in green, EdU positive in red, nucleus in blue). Scale bar, 200 μm.


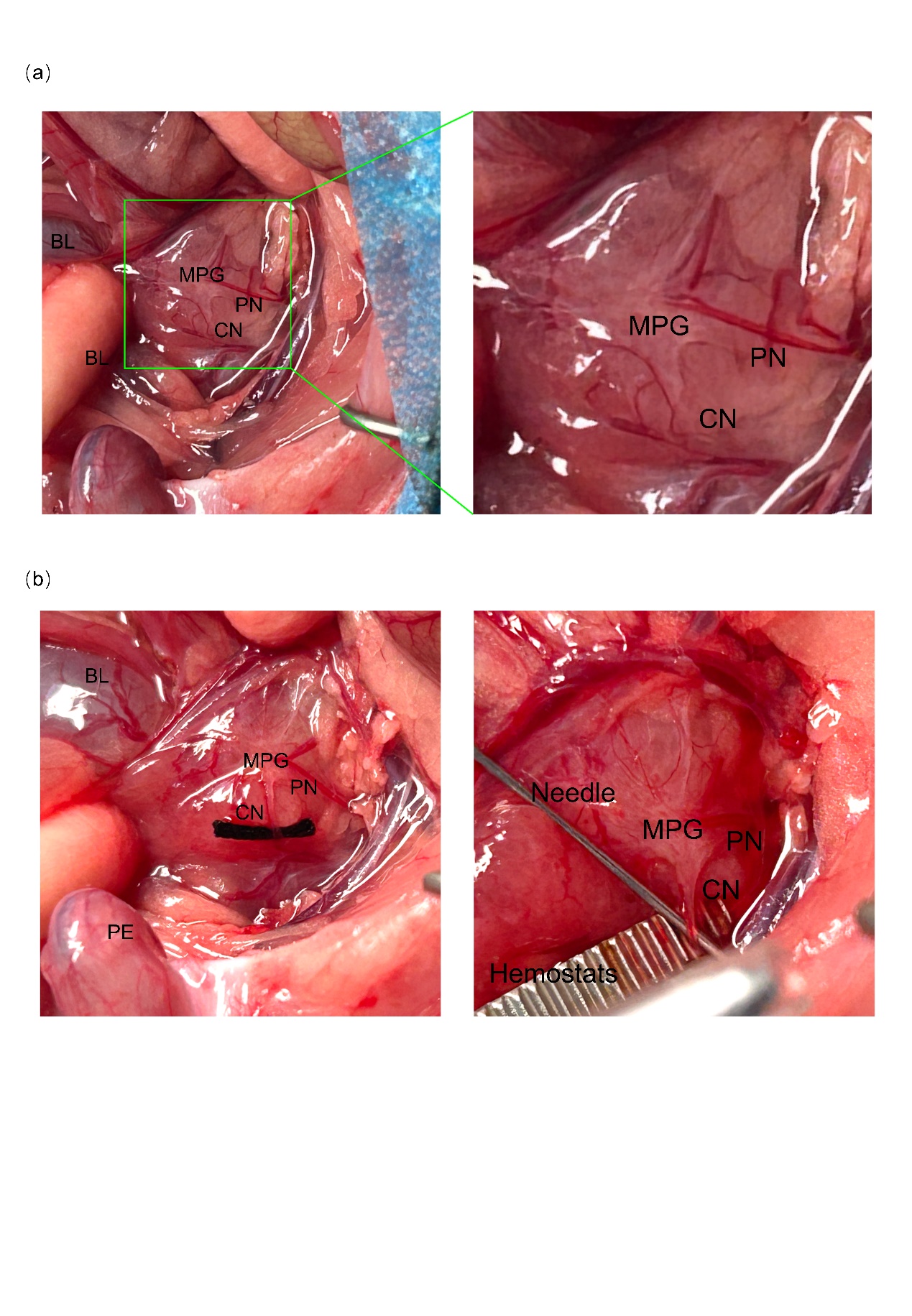


**Fig. S5** (a) Representative image of MPG and CN. (b) Representative image of CN anatomical separation (over the black suture) and the "hemostat tip-syringe needle-nerve-hemostat tip" sandwich structure for BCNI model construction (BL, bladder; PE, penis; MPG, main pelvic ganglion; CN, cavernous nerve; PN, pelvic nerve).


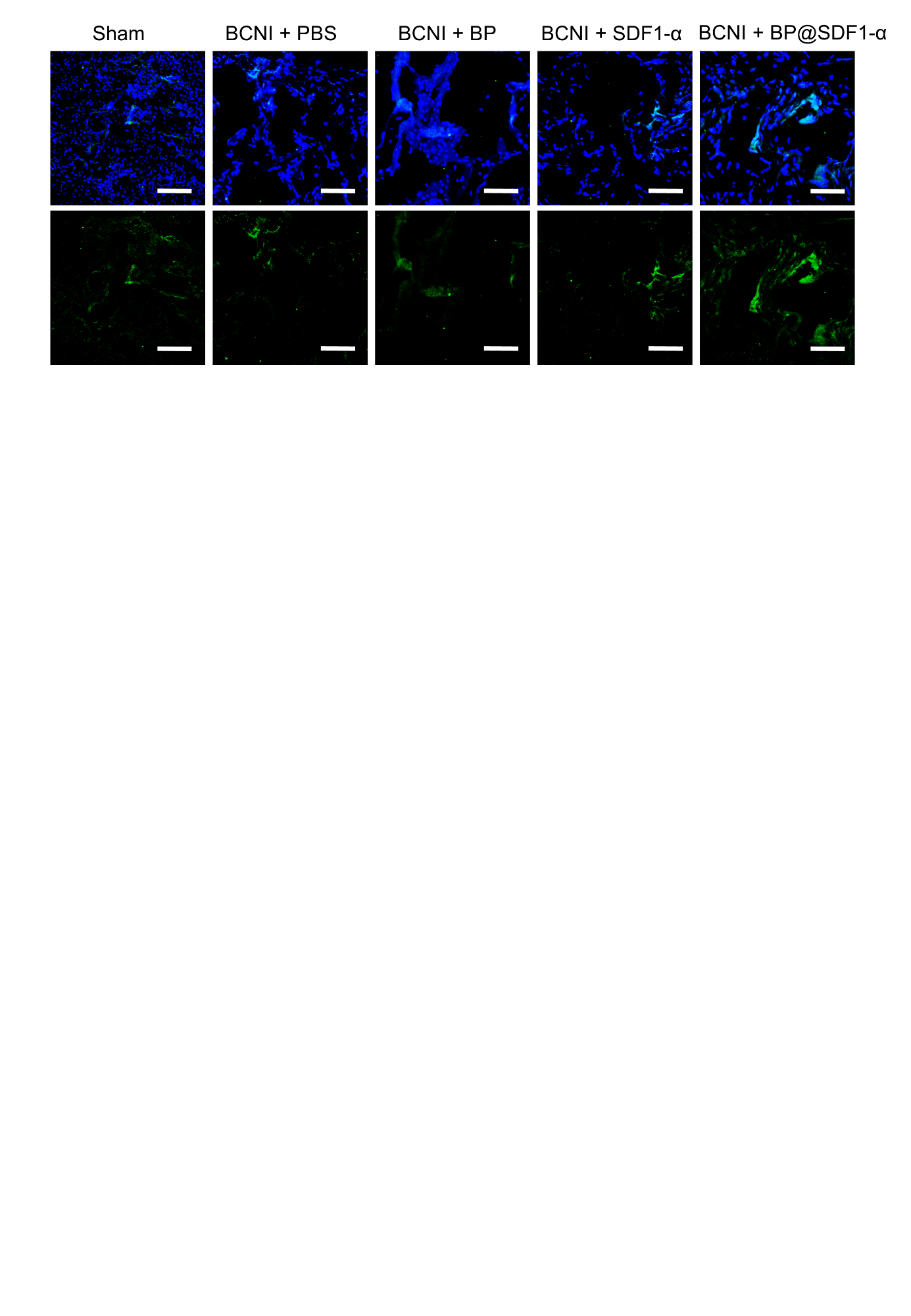


**Fig. S6** The expression of SDF1-α in penis tissues was assessed by immunofluorescence staining and visualized under a laser confocal microscope (SDF1-α in green, nucleus in blue). Scale bar, 200 μm.


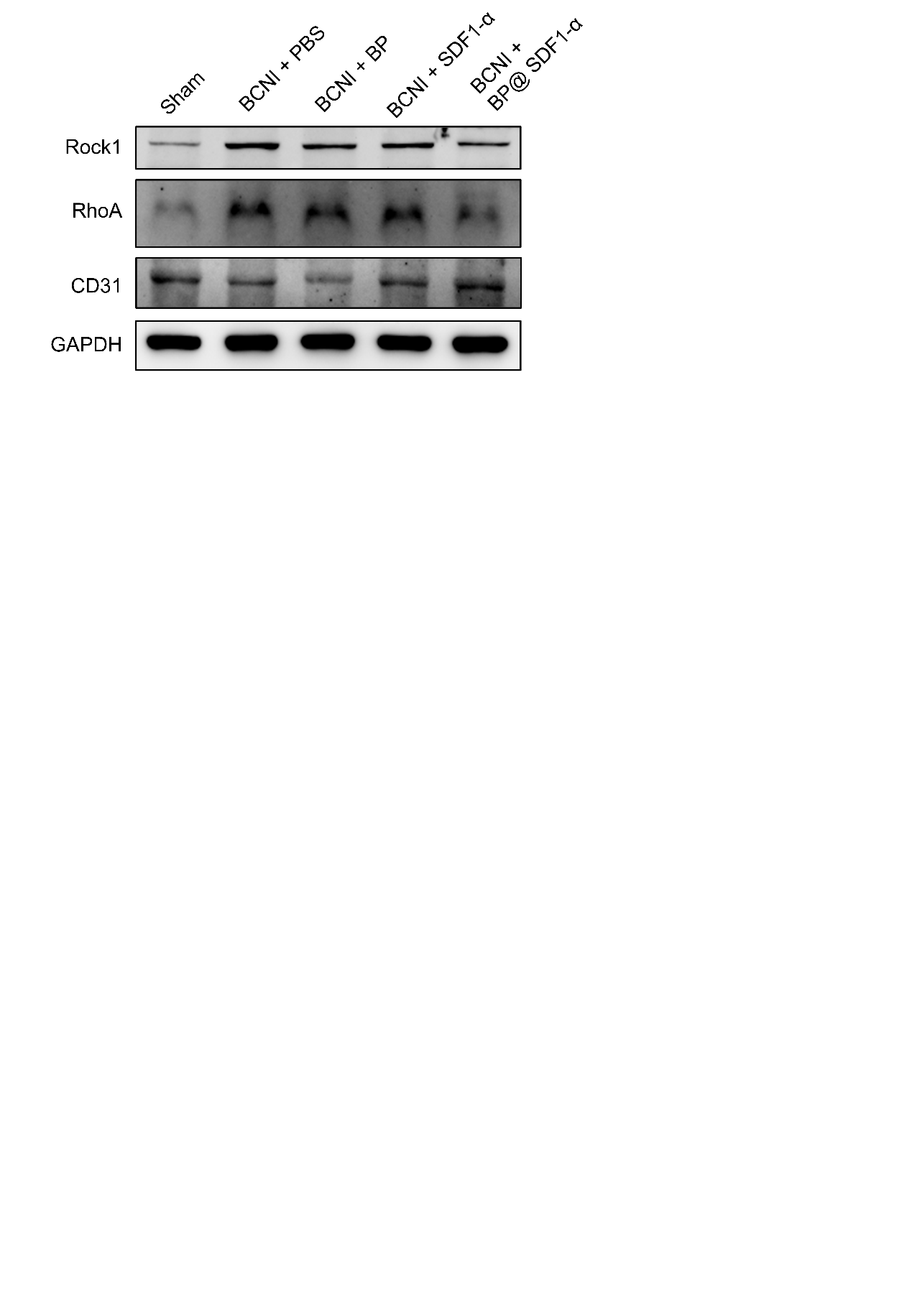


**Fig. S****7** The protein expression level of Rock1, RhoA, and CD31 in penis tissues was detected by Western Blotting.
